# Supplementary material for: Effects of age on non-communicable disease risk factors among Nepalese adults
Source: PLoS One. 2023 Jun 2;18(6):e0281028. doi: 10.1371/journal.pone.0281028 (PMC10237426; doi:10.1371/journal.pone.0281028)
Supplement: S1 File — (DOCX) [file pone.0281028.s001.docx]

# **Supporting information for the manuscript entitled “effects of age on non-communicable diseases risk factors among Nepalese adult population”**

**Supplementary information 1(S1): Definition and measurement of variables under study**

**Sociodemographic characteristics**

**Age**

*Age was categorized into three groups:*

- less than 35 years (Young)
- 35-59 years (Middle aged)
- 60 years and above/60+ years (Elderly)

***Sex:*** Male or female gender of the respondents

**Ethnicity**

*Ethnic groups of the respondents were classified into five categories:*

- Dalits: so-called non-touchable ethnic groups
- Disadvantaged janjati: disadvantaged ethnic groups and terai caste,
- Religious minorities: Minor ethic groups including Muslims
- Advantaged janjati
- Upper caste

**Level of educational attainment**

*The educational attainment of the respondents has been classified into four categories:*

- Below primary: No formal education or less than five years of education
- Primary completed: completed 8^th^ grade of formal education
- Secondary level (12 yr.): completed 12^th^ grade of formal education
- University education: completed at least bachelor level education

**Current occupation**

*The occupation of the respondents has been classified into five categories:*

- Unemployment: Not working at all
- Employment: Government employees, those working in the formal sector
- Homemaker: those working as housewives
- Students: those who are studying
- Others: Non paid workers, retired and those working in the non-formal sector,

**Urban-Rural Location**

- Rural: Respondents currently residing in the rural municipality for the last six months
- Urban: Respondents currently residing in metropolitan city/Sub metropolitan city, and municipality for the last six months

**Wealth Index**

*The wealth index of the households had been calculated by considering the type of materials used for the household roof, access to electricity, possession of consumer goods (e.g. Television, Mobile, Radio), and means of transportation (bicycle, motorbike, car), etc. Wealth quantiles (WQ) had been calculated by using Principal Component Analysis (PCA). For our analysis, these five categories of wealth index have been recoded into three classes:*

- Lower wealth index: Lowest and Second wealth quantiles
- Middle wealth index: Middle wealth quantiles
- Higher wealth index: Fourth and highest wealth quantiles

**Behavioral risk factors**

**Tobacco use:**

Prevalence of tobacco use has been assessed by asking if the participant currently smokes any tobacco products (cigarettes, bidis, cigars, pipes, hukkah, or tamakhus) or uses any smokeless tobacco products (snuffs, chewing tobacco, khaini, surti, gutkha, etc) daily. For this study, daily smoking has been included as study variable.

**Alcohol consumption:**
Prevalence of alcohol consumption has been assessed by asking if the participant had consumed alcohol (beer, wine, spirits, fermented cider or Jaand, Chhyang, Raksi, Aila, or Tongwa) within the past 12 months.

**Insufficient physical activity:**

Physical activity was calculated considering the type of physical/labor work, transport (walking, cycling), and leisure/sports activities. Those respondents who were engaged in less than 75 minutes of vigorous physical activity or less than 150 minutes of moderate physical activity per week were categorized as insufficient physical activity.

**Insufficient consumption of fruits and vegetables:**

The amount of fruit and vegetables consumed per day has been recorded of all respondents in terms of servings per day. A serving of fruit/vegetables equates to one small fruit, ½ a cup of raw vegetables, one cup of leafy greens, or one banana (1 servings = 80 gram approximately)

As the study measured an average of 2 servings (160 gm) per day, consumption of 2 or less than 2 servings per day had been considered as insufficient consumption of fruits and vegetables. (Note: The WHO recommendation for sufficient consumption of fruits and vegetables requires at least five servings (400 grams) per day.)

**Biological risk factors**

**Overweight/obesity**

Height (in centimeters) and weight (in kilograms) were measured using portable standard stature tape (SECA, Germany) and portable digital weighing scales (SECA, Germany), respectively. Height was taken while the individual stood in an upright position on a flat surface. Body Mass Index (BMI) was computed based on height and weight measurement and categorized into the following four categories:

- Underweight: BMI less than 18.5 kg/m^2^
- Normal weight: BMI 18.5-24.9 kg/ m^2^
- Overweight: BMI 25-30 kg/ m^2^
- Obesity: BMI more than 30 kg/ m^2^

*In addition, a binary variable was constructed, indicating high body weight:*

- High body weight includes overweight and obesity: BMI 25 more than 25 kg/m^2^

**Raised blood pressure (measured as hypertension)**

Blood pressure (BP) was measured using an OMRON™ digital automatic BP monitor with a universal-sized cuff. The mean of systolic and diastolic blood pressure was estimated based on the three readings taken in three minutes intervals. Respondents having systolic blood pressure more than 140 mmHg and/or diastolic blood pressure more than 90 mmHg and/or currently under medication for hypertension have been categorized as ‘Raised Blood Pressure’ and measured as ‘hypertension’

**Raised blood glucose (measured as hyperglycemia)**

Fasting blood glucose measurements were taken a day after they were confirmed to have had an overnight fast. Serum Glucose was measured by dry chemistry using a CardioCheck PA analyzer for point-of-care testing. A drop of capillary blood from a finger prick was used to test for glucose levels using a digital meter (ACCUCHECK™ glucometer and test strips). Respondents with fasting blood glucose of more than 125 mg/dl and/or respondents currently taking hypoglycemic drugs have been categorized as ‘raised blood glucose’ and measured as ‘hyperglycemia’

**Raised total cholesterol (measured as hyperlipidemia)**

Total serum cholesterol has been measured by using the same sample drawn for the fasting glucose. Total cholesterol in serum was measured by dry chemistry using a CardioCheck PA analyzer for point-of-care testing. A drop of capillary blood from a finger prick was used to test for total cholesterol using a digital meter (ACCUCHECK™ glucometer and test strips). Respondents having total cholesterol of more than 190 mg/dl and/or respondents currently under medication for raised total cholesterol had been categorized as ‘raised total cholesterol’ and measured as ‘hyperlipidemia’
