# Supplementary material for: Effects of age on non-communicable disease risk factors among Nepalese adults
Source: PLoS One. 2023 Jun 2;18(6):e0281028. doi: 10.1371/journal.pone.0281028 (PMC10237426; doi:10.1371/journal.pone.0281028)
Supplement: S2 File — (DOCX) [file pone.0281028.s002.docx]

**Supplementary information 2 (S2): Weighted proportion of sociodemographic variables indicating the true representation of the study population**

# Table S2: Sociodemographic characteristics of the study population stratified by age groups (weighted proportions, considering the survey design)

| **Demographic  variables** | **Census report (%)** | **Aggregate frequencies**  **(weighted %)**  **(n=5593)** | **stratified frequencies (weighted percentage)** | | | **p-value *** |
| --- | --- | --- | --- | --- | --- | --- |
|  |  |  | **Below 35 years**  **(n=2196)  [census report %]** | **35-59 Years**  **(n=2705)**  **[census report %]** | **60 years and above**  (**n=692)**  **[census report %]** |  |
| **Gender** |  |  |  |  |  |  |
| Men | 48.9 | 1998(47.2) | 647(45.6)  [45.8] | 1040(48.5) [48.3] | 311(49.6) [49.4] | **0.23** |
| Women | 51.1 | 3595(52.7) | 1549(54.4)  [54.1] | 1665(51.5) [51.6] | 381(50.3) [50.5] |  |
| **Ethnic Group** |  |  |  |  |  |  |
| Dalits | 12.6 | 766(11.8) | 347(11.1) | 326(9.6) | 93(10.2) | **0.14** |
| Disadvantaged janjati | 34.7 | 1640(34.1) | 634(35.3) | 809(36.2) | 197(36.9) |  |
| Religious minorities | 4.2 | 174(5.6) | 83(6.5) | 76(4.2) | 15(3.6) |  |
| Advantaged janajati | 15.5 | 899(14.0) | 327(13.5) | 458(15.8) | 114(15.6) |  |
| Upper caste | 34.1 | 2114(34.2) | 805(33.4) | 1036(33.9) | 273(33.4) |  |
| **Level of education** |  |  |  |  |  |  |
| Below primary |  | 2792(37.8) | 580(20.5) | 1632(58.5) | 580(82.7) | **<0.001** |
| Primary completed |  | 1051(19.1) | 520(22.6) | 463(18.1) | 68(11.6) |  |
| Secondary level(12 yr.) |  | 1573(39.2) | 990(52.2) | 547(21.1) | 36(5.1) |  |
| University education |  | 176(3.7) | 106(4.5) | 63(2.0) | 7(0.5) |  |
| **Occupation** |  |  |  |  |  |  |
| Unemployment |  | 273(6.0) | 100(5.7) | 102(5.3) | 71(11.4) | **<0.001** |
| Employment |  | 1353(24.7) | 425(19.9) | 815(33.4) | 113(17.6) |  |
| Students |  | 402(14.3) | 394(25.9) | 5(0.1) | 3(0.2) |  |
| Home makers |  | 3142(45.7) | 1095(37.7) | 1585(53.1) | 462(64.4) |  |
| Others |  | 417(9.3) | 180(10.5) | 195(7.9) | 42(6.2) |  |
| **Location** |  |  |  |  |  |  |
| Rural | 34 | 2133(37.2) | 824(37.9) | 1052(36.5) | 257(35.7) | **0.63** |
| Urban | 66 | 3460(62.8) | 1372(62.0) | 1653(63.4) | 435(64.2) |  |
| **Strata** |  |  |  |  |  |  |
| Rural Municipality |  | 2133(37.2) | 825(37.8) | 1052(36.3) | 257(35.7) | **0.78** |
| Sub-/Metropolitan City |  | 705(8.9) | 282(9.1) | 346(8.7) | 77(8.0) |  |
| Municipality |  | 2755(53.8) | 1090(52.8) | 1307(54.7) | 358(56.1) |  |
| **Wealth Index** |  |  |  |  |  |  |
| Lower Wealth Index |  | 2715(49.0) | 1035(46.4) | 1320(47.9) | 360(51.3) | **0.62** |
| Middle Wealth Index |  | 949(16.3) | 384(17.2) | 451(16.1) | 114(15.5) |  |
| Upper Wealth Index |  | 1929(34.7) | 777(36.2) | 934(35.8) | 218(33.0) |  |

* p-value of the Chi-square test for differences between age groups
