# Supplementary material for: Effects of age on non-communicable disease risk factors among Nepalese adults
Source: PLoS One. 2023 Jun 2;18(6):e0281028. doi: 10.1371/journal.pone.0281028 (PMC10237426; doi:10.1371/journal.pone.0281028)
Supplement: Attachment — Submitted filename: Review comments.docx [file pone.0281028.s003.docx]

**Review comments**

**The authors have done an interesting study to see how the risk factors of NCDs vary with age which will add some knowledge in the existing information of the NCDs in Nepal. The authors can revise the following information in their submission.**

1. In the supporting information S1, the educational attainment of respondents has been classified as : Illiterate, Primary level, Secondary Level and University level which does not match with that of the names used in the table under supporting information S2 that has “below primary”, “primary completed”, “secondary level” and “University Education”. Please make consistent where necessary

2. Does “others” under the heading “occupation” refer to “Non paid workers and retired” as stated in S1? Please look for similar inconsistencies.

3. Under ethical approval heading in the main document, please write full form of NHRC and LMU when they are used for the first time as an acronym in the main document.

4. Fix some typo; change cast to caste in S1.

5. The age has been broadly categorized into three groups i.e. <35 years, 35-59 years and >=60 years which seems to be a major limitation of the study as this broad categorization can not give a reliable estimate of how age modifies the effect of health behavior on biological risk factors. This creates a problem particularly for analysis of women’s data because of the confounded effect of menopausal symptoms and hormonal changes during the 40’s among women. The authors might consider adding a limitation on why they could not measure age on a continuous scale to measure its effect on non-communicable disease disk factors.
