# Supplementary material for: Effects of age on non-communicable disease risk factors among Nepalese adults
Source: PLoS One. 2023 Jun 2;18(6):e0281028. doi: 10.1371/journal.pone.0281028 (PMC10237426; doi:10.1371/journal.pone.0281028)
Supplement: Attachment — Submitted filename: 4.21 Responses for Reviewer .docx [file pone.0281028.s004.docx]

21 April, 2023

To

The Academic Editor

PLoS One

Ref: Responses to the review comments on PONE-D-23-01133

Dear Sir/Madam

I would like to express my sincere thanks for accepting our scientific work entitled “Effects of age on non-communicable disease risk factors among Nepalese adults” in your esteemed journal. On behalf of all co-authors, I would like to acknowledge the review comments and suggestion on the manuscript submitted for publication. We tried our best effort to incorporate the comments and presented here in the table below.

Thank you for your cooperation and support

With best regards

Bhim Prasad Sapkota

Corresponding author

**Point by point responses in tabular form:**

| **SN** | **Comments from Reviewers/Editor** | **Response from the Author** |
| --- | --- | --- |
| 1. | In the supporting information S1, the educational attainment of respondents has been classified as: Illiterate, Primary level, Secondary Level and University level which does not match with that of the names used in the table under supporting information S2 that has “below primary”, “primary completed”, “secondary level” and “University Education”. Please make consistent where necessary | Thank you for your comment.  The information in the supplementary files (S1 and S2) has been revised and the following terms have been included:  Primary Incomplete  Primary Complete  Secondary (12 yr.)  University |
|  | Do “others” under the heading “occupation” refer to “Non paid workers and retired” as stated in S1? Please look for similar inconsistencies. | Thank you for making this important suggestion.  This has been revised to ensure consistency, and checks have been undertaken throughout the manuscript.  As mentioned in the operational definition of the occupation, “Non paid workers and retired” have been included in the “Others” category. |
| 3 | Under ethical approval heading in the main document, please write full form of NHRC and LMU when they are used for the first time as an acronym in the main document. | Thank you for pointing this out, this has been revised accordingly. |
| 4 | Fix some typo; change cast to caste in S1. | Thank you, spelling errors have been corrected throughout. |
| 5 | The age has been broadly categorized into three groups i.e. <35 years, 35-59 years and >=60 years which seems to be a major limitation of the study as this broad categorization cannot give a reliable estimate of how age modifies the effect of health behavior on biological risk factors. This creates a problem particularly for analysis of women’s data because of the confounded effect of menopausal symptoms and hormonal changes during the 40’s among women. The authors might consider adding a limitation on why they could not measure age on a continuous scale to measure its effect on non-communicable disease disk factors. | Thank you for emphasizing this limitation.  We have addressed this limitation in the revised version of the manuscript (Page 11, line 8-11). |
| 6 | Abstract word count is exceeding the limit as per journal guideline. | Thank you! We reduced the number of words in the abstract to 296. |
| 7 | Please remove the bullet points from the discussion. Paragraphs would be better. | The discussion part has been revised; the bullet points have been replaced by paragraphs. |
| 8 | Was there any effect of age on smoking? | Thank you for this relevant question.  The effect of age on smoking is described under Results, under the subheading Prevalance of Behavioral and biological risk factors. |
| 9 | Plausible reason for hyperlipidemia increasing with age but not with smoking in this study needs to be explained. | Thank you for this suggestion.  We describe a possible reason for the lack of an association between hyperlipidemia and smoking under Results, under the sub heading Effects of health behaviors on biological risk factors and their associations with age section: “The effect of daily smoking on hyperlipidemia was not statistically significant, which could be due to the masking effect of age on hyperlipidemia.” |
| 10 | The references (esp. #1-3) used in the introduction are older which could be replaced with newer latest evidence and edition of the same or similar reports. E.g., you could use the progress monitor report available for 2022 - Noncommunicable Diseases Progress Monitor 2022 ([who.int](http://who.int/)));  furthermore, it’s better to use the global status report rather than citing a big statement about the situation of NCDs from the global action plan for prevention and control of NCDs and that is older as well; and the latest one of the global status report could also be used. | Thank you for suggesting these more recent publications. Recent publications, e.g. GBD result 2019 and Progress Monitoring of NCDs 2022 are now included in the revised version of the manuscript. |
| 11 | Check if the ref #4 is listed correctly in the references list. Please check other references as well and make sure they are complete. For e.g., # 9 looks bit incomplete. Other references also seem to have been written incorrectly (e.g. 12, 13); Nepal STEPS 2019 report could be referenced more appropriately (also it has been listed in the ref list twice). and so on… hence please check all the references thoroughly. | Thank you for identifying these errors.  All references were checked for accuracy, and corrections made where necessary. |
| 12 | In the beginning of second para the information cited with ref #9 looks to be old (2009 report?). There are several latest evidence available for this as well. | Thank you, this information has now been updated using the latest information (NCDs fact sheet published by WHO in September 2022) |
| 13 | For reporting the latest life expectancy of Nepal you could either use world bank data or GBD 2019 data as reference. | Thank you for this constructive feedback.  For the latest life expectancy figures, we used the Nepal GBD study 2019. |
| 14 | In the behavioral risk factors, why only daily smoking and no other forms or tobacco use was considered including non-daily smoking? | This is a valid question.  The literature suggests that among the different variables available to measure tobacco consumption, daily smoking represents the most reliable measure of intense exposure to tobacco smoke. We have included this concern as limitation of the study as follows: “Given the large number of variables included in our analysis, we had to limit ourselves to one measure of tobacco and chose the one that would likely show the greatest effect”. |
| 15 | Biological risk factors – better to specify cholesterol as total cholesterol. | ‘Cholesterol’ has now been replaced by “total cholesterol” wherever necessary. |
| 16 | In the section prevalence of behavioral and biological risk factors the second line talks about tobacco among women in different age groups but compares at the end with single group of men which looks little irrelevant comparison, please clarify/amend. | Thank you,  the sentences have been rephrased as follows: … “…the increase was far stronger in women, ranging from 1% prevalence for the youngest to 12% among the middle aged and 23% for the oldest age group. Among men, prevalence of daily smoking increased from 17% among the youngest to 24% among the middle aged and 30% in the elderly.” |
| 17 | In the same section, in second para you suddenly start using hypertension and hyperlipidemia while your variables are raised BP and raised total cholesterol. Suggest to stick to original variables, and also hyperlipidemia may not be the right choice of word since we are just talking about total cholesterol and not other cholesterols. And follow the use of same name for risk factors in rest of the manuscript. | Thank you for pointing out these inconsistencies.  Operational definitions of study variables have been revised as  **Hypertension**=Raised BP and those taking antihypertensive drugs.  **Hyperglycemia=**Raised blood glucose and those taking hypoglycemic drugs  **Hyperlipidemia**=Raised total cholesterol and those taking lipolytic drugs  We now use the correct terms throughout the revised manuscript. |
| 18 | In addition, while describing the results by sex I would always add age disaggregated description (which you have done in some places but not in all, please follow this to justify your title and main objective of the study) since that is your main variable of interest. | Thank you for this constructive feedback.  We tried to present the age disaggregated information in relevant section. |
| 19 | The difference of prevalence of risk factors in general is already described by the report and manuscript of the STEPS survey. Please follow the same in the third para of this section – on the treatment seeking behavior part. | Thank you for this relevant suggestion.  Under Results, under the sub-section Prevalence of behavioral and biological risk factors, we added the following sentence: “The treatment rate was lower among women compared to men, and lowest in women and men aged 60 years and older.” |
| 20 | In the last two sections of results you have attempted to describe the effects of age, which is commendable and is the beauty of this manuscript. However, this could be further beautified by being more consistent and coherent in the description. Try to ensure you describe the relationship or effects of other variables on your dependent variables and then describe what and how age has affected that. You have done in some part, but readers will love it if you do that consistently and coherently. Try to read it once from this angle, and this will be done easily. | Thank you for this important comment.  In the 3^rd^ paragraph and subsequently (page 7) we have described the effects of sociodemographic variables on behavioral risk factors (based on figures of table 3).  But with the intention to keep the result section clear and concise, we provided less description about the effect of other variable on NCD risk factors. We focused on the effects of age on NCD risk factors in line with the objective of our study.  We hope the research question has been clearly answered by the descriptions in result section. |
| 21 | **Strengths and limitations** The last line about biological measures look to be written as limitation, but I believe you meant that is the strength, so revise that to reflect that you wanted to say that it was one of the strengths of the study. | Thank you, and you are right. This has now been moved to the first paragraph and has been re-written as a strength. |
| 22 | **Conclusion** Your conclusion is more generic towards health systems improvement in most of the statements except few where you have touched the age part. Readers would love to see some more specific conclusion/recommendation based on your specific significant results of your analysis of age effect. That would make the conclusion part even better and precise. | Thank you for your nice feedback.  As per your suggestion, the conclusion has been revised to make it consistent with the specific findings of the study |
| 23 | **Abstract**  I recommend revising the conclusion part in the summary as well after you are done revising your paper as above. | Thank you for comments.  The conclusion part in the abstract has been rephrased as follows: “It is necessary to diagnose and treat biological risk factors, in younger age groups before they manifest as NCDs. Similarly, behavior change interventions need to target these younger age groups to reduce the risk of NCDs later in life” |
